# Metabolic changes in the plasma of mild Alzheimer’s disease patients treated with Hachimijiogan

**DOI:** 10.3389/fphar.2023.1203349

**Published:** 2023-06-12

**Authors:** Mosaburo Kainuma, Shinobu Kawakatsu, Jun-Dal Kim, Shinji Ouma, Osamu Iritani, Ken-Ichiro Yamashita, Tomoyuki Ohara, Shigeki Hirano, Shiro Suda, Tadanori Hamano, Sotaro Hieda, Masaaki Yasui, Aoi Yoshiiwa, Seiji Shiota, Masaya Hironishi, Kenji Wada-Isoe, Daiki Sasabayashi, Sho Yamasaki, Masayuki Murata, Kouta Funakoshi, Kouji Hayashi, Norimichi Shirafuji, Hirohito Sasaki, Yoshinori Kajimoto, Yukiko Mori, Michio Suzuki, Hidefumi Ito, Kenjiro Ono, Yoshio Tsuboi

**Affiliations:** ^1^ Department of Japanese Oriental Medicine Graduate School of Medicine and Pharmaceutical Sciences, University of Toyama, Toyama, Japan; ^2^ Aizu Medical Center, Department of Neuropsychiatry, Fukushima Medical University, Aizuwakamatsu, Japan; ^3^ Department of Research and Development, Division of Complex Biosystem Research (CBR), Institute of National Medicine (INM), University of Toyama, Toyama, Japan; ^4^ Department of Neurology, School of Medicine, Fukuoka University, Fukuoka, Japan; ^5^ Department of Geriatric Medicine, Kanazawa Medical University, Ishikawa, Japan; ^6^ Translational Neuroscience Center, Graduate School of Medicine, International University of Health and Welfare, Tochigi, Japan; ^7^ Department of Neuropsychiatry, Graduate School of Medical Sciences, Kyushu University, Fukuoka, Japan; ^8^ Department of Neurology, Graduate School of Medicine, Chiba University, Chiba, Japan; ^9^ Department of Psychiatry, Jichi Medical University, Tochigi, Japan; ^10^ Second Department of Internal Medicine, Division of Neurology, Faculty of Medical Sciences, University of Fukui, Fukui, Japan; ^11^ Department of Medicine, Division of Neurology, Showa University School of Medicine, Tokyo, Japan; ^12^ Department of Neurology, Wakayama Medical University, Wakayama, Japan; ^13^ Department of General Medicine, Oita University Faculty of Medicine, Oita, Japan; ^14^ Department of Internal Medicine, Wakayama Medical University Kihoku Hospital, Wakayama, Japan; ^15^ Department of Dementia Medicine, Kawasaki Medical School, Okayama, Japan; ^16^ Department of Neuropsychiatry, Graduate School of Medicine and Pharmaceutical Sciences, University of Toyama, Toyama, Japan; ^17^ Department of General Internal Medicine, Kyushu University Hospital, Fukuoka, Japan; ^18^ Department of Clinical Research Promotion, Kyushu University Hospital, Fukuoka, Japan; ^19^ Department of Rehabilitation, Fukui Health Science University, Fukui, Japan; ^20^ Department of Neurology, Kanazawa University Graduate School of Medical Sciences, Ishikawa, Japan

**Keywords:** metabolomic analysis, Alzheimer’s disease, hachimijiogan, aspartic acid, kampo

## Abstract

**Background:** Alzheimer’s disease (AD), the most prevalent form of dementia, is a debilitating, progressive neurodegeneration. Amino acids play a wide variety of physiological and pathophysiological roles in the nervous system, and their levels and disorders related to their synthesis have been related to cognitive impairment, the core feature of AD. Our previous multicenter trial showed that hachimijiogan (HJG), a traditional Japanese herbal medicine (Kampo), has an adjuvant effect for Acetylcholine estelase inhibitors (AChEIs) and that it delays the deterioration of the cognitive dysfunction of female patients with mild AD. However, there are aspects of the molecular mechanism(s) by which HJG improves cognitive dysfunction that remain unclear.

**Objectives:** To elucidate through metabolomic analysis the mechanism(s) of HJG for mild AD based on changes in plasma metabolites.

**Methods:** Sixty-seven patients with mild AD were randomly assigned to either an HJG group taking HJG extract 7.5 g/day in addition to AChEI or to a control group treated only with AChEI (HJG:33, Control:34). Blood samples were collected before, 3 months, and 6 months after the first drug administration. Comprehensive metabolomic analyses of plasma samples were done by optimized LC-MS/MS and GC-MS/MS methods. The web-based software MetaboAnalyst 5.0 was used for partial least square-discriminant analysis (PLS-DA) to visualize and compare the dynamics of changes in the concentrations of the identified metabolites.

**Results:** The VIP (Variable Importance in Projection) score of the PLS-DA analysis of female participants revealed a significantly higher increase in plasma metabolite levels after HJG administration for 6 months than was seen in the control group. In univariate analysis, the aspartic acid level of female participants showed a significantly higher increase from baseline after HJG administration for 6 months when compared with the control group.

**Conclusion:** Aspartic acid was a major contributor to the difference between the female HJG and control group participants of this study. Several metabolites were shown to be related to the mechanism of HJG effectiveness for mild AD.

## Introduction

The cognitive and memory impairment of dementia patients has been shown to lead to negative changes in behavior and activities of daily life (Cipriani et al., 2020). Currently, more than 55 million people suffer from dementia worldwide, with an incidence of about 10 million each year. Alzheimer’s disease (AD), the most prevalent form of dementia, is a debilitating, progressive form of neurodegeneration that accounts for 60%–70% of this disease that creates an enormous burden on public health systems around the world (World health Organization, 2023).

Numerous therapies have been developed in an attempt to alleviate or cure symptoms (Jeon et al., 2019). Notably, aducanumab was recently approved for mild AD (Billy et al., 2021); however, there is currently no available treatment that is an effective cure. The discovery and introduction of new therapeutic agents and strategies for AD are urgently needed.

“Kidney^[^™^]^ deficiency” is one of the key pathologies of Kampo medicine, defined as a pattern characterized by an insufficient amount of essential qi of kidney. Hachimijiogan (HJG) is a traditional Japanese (herbal) medicine (Kampo) composed of eight herbs that is prescribed for the treatment of “Kidney^[^™^]^ deficiency” (Terasawa, 1993; Kainuma et al., 2022). It is effective for various symptoms common to members of the older population, such as numbness, nocturia, lower back pain and coldness in the legs, and is used for cases of impotence, nephritis, diabetes, sciatica, bladder catarrh, lumbago, prostatic hypertrophy, and hypertension (Nakae et al., 2018). In Kampo, cognitive impairment is considered to be a sign of “Kidney^[^™^]^ deficiency”. We previously demonstrated the effectiveness of HJG for AD both *in vivo* and *in vitro*: improvement was seen in the spatial memory impairment of AD model rats and in cognitive function via cAMP response element binding protein (CREB) (Kubota et al., 2017; Moriyama et al., 2017). Although not conclusive, we reported in a clinical trial that HJG has an adjuvant effect for acetylcholinesterase inhibitors (AChEIs) and that it delays the deterioration of the cognitive dysfunction of mild AD patients (Kainuma et al., 2022). However, there are aspects of the molecular mechanism(s) by which HJG improves cognitive dysfunction that remain unclear.

Metabolomics is a system-biology technology that can be used to monitor the dynamic changes of endogenous small-molecule metabolites (Nicholson et al., 2002). Several blood metabolic biomarker studies of AD have reported that changes in phospholipids, polyamines, and amino acids have potential for use in diagnosis (Chouraki et al., 2017; Huo et al., 2020; Lin et al., 2019b; Olazarán et al., 2015; Orešič et al., 2011; Ozaki et al., 2022; Peña-Bautista et al., 2019; Shao et al., 2020; Trushina and Mielke, 2014; Wan et al., 2020; Wilkins and Trushina, 2018). Kampo medicines are multi-component, multi-targeted drugs that act on various targets in the body. To understand the complex actions of Kampo medicines, it is important to extensively study the biological reactions they induce and the relation of the components to these reactions. Because it has been shown to be a useful tool for elucidating complex pharmacological actions and evaluating the effects of Kampo medicines on the living body (Kitagawa et al., 2018; Yamashita et al., 2021; Kobayashi et al., 2022), comprehensive metabolomic analysis was used in this study to determine the mechanism(s) of the effectiveness of HJG for mild AD.

## Methods

The data analyzed in this study was from our previous open-label, randomized, multicenter, control trials (Kainuma et al., 2020; Kainuma et al., 2022).

### Ethics

The study was done in accordance with the principles of the Declarations of Helsinki and Tokyo and approved by the Kyushu University Hospital Clinical Research Review Board (CRB) (Fukuoka, Japan) (CRB approval number: KD 2019001). All participants gave written, informed consent.

### Study design

This study started 2 August 2019 at three sites in Japan. Unfortunately, recruitment was delayed because of the COVID-19 pandemic, but we adjusted and were able to add 11 institutions by January of 2021. The study finished on 31 March 2022. In brief, our protocol was done with mild AD patients who met all the inclusion criteria:

1) Age: ≥50 to <85 years old, 2) Mild Alzheimer’s disease (MMSE ≥21), 3) Taking the same dose of Donepezil, Galantamine, or Rivastigmine for more than 3 months, 4) Not taking Memantine, and 5) Written informed consent. Patients were excluded who were taking Kampo Medicine other than HJG for more than 3 months or who had a change in drug dosage that could affect the progression of cognitive function during the 3 months. The other major exclusion criteria are as follows: 1) Kidney dysfunction (eGFR<30 mL/min/1.73 m^2^); 2) AST or ALT>100 IU/L; 3) Complication with gastric ulcer, bronchial asthma, or epilepsy; and 4) Judged by doctors not to be suited for study, such as having serious complications.

Participants were divided into an AChEI plus HJG group and an AChEI alone group to compare the effectiveness and safety of the addition of HJG. In addition to AChEI, the HJG group took 2.5 g of HJG extract 3 times/day (TSUMURA hachimijiogan Extract Granules for Ethical Use: TJ-7, HJG, Tokyo Japan), the usual adult daily dose. Randomization was done with the Randomization Module of Research Electronical Data Capture (RED Cap). A computer-generated list of random numbers was transferred to RED Cap for block randomization by age and sex, then the participants were randomly assigned at a 1:1 ratio. Participants, physicians and data evaluators were aware of the allocation group after randomization, but outcome evaluators were blinded.

The primary outcome was the change of the Cognitive Component of the Alzheimer’s Disease Assessment Scale—Japanese Version (ADAS-Jcog) from baseline to 6 months, with assessments done at baseline and after 3 and 6 months. Blood tests were done at the same time points for the analyses of the metabolome, with samples collected at least 4 hours after breakfast. Plasma was separated by centrifugation at 2000 f for 25 min at 4°C, immediately stored at −80°C, and kept frozen until use.

### Metabolomics analysis

We analyzed the plasma metabolome using gas chromatography-tandem mass spectrometry (GC-MS/MS; GCMS-TQ8040, Shimadzu, Kyoto, Japan) and liquid chromatography-tandem mass spectrometry (LC-MS/MS; Nexera X2 system connected with LCMS-8050, Shimadzu). We extracted, measured, and analyzed both hydrophilic and hydrophobic metabolites, including lipid mediators and phospholipids, in accordance with previously reported methods (Kitagawa et al., 2018; Yamashita et al., 2021).

### Statistical analysis

The search for the HJG biomarkers was done through analysis of covariance of the change in metabolites from baseline, with the treatment group and baseline values as covariates. Continuous variables are expressed as the mean value and standard deviation.

A two-tailed test at the 5% level of significance was done for the statistical analyses. Two-tailed interval estimation was done with a 95% confidence interval (CI) and corresponding *p* values (with significance defined as *p* < 0.05). A *t*-test was used for comparison of the metabolomics of the HJG and control groups at each visit, with *p* < 0.05 representing a significant difference. JMP (Ver. 16, SAS Institute Japan Ltd.) was used for these analyses. In addition, to maximize class discrimination necessary for the identification discovery of potential metabolic biomarkers of between the HJG and Control groups, a supervised model of potential least squares discriminant analysis (PLS-DA) was applied. Variable importance in the projection (VIP) values were used in the PLS-DA model to estimate the discriminatory power of each variable for separation of the groups. The features, and variables with VIP values > 1 were considered important. The validation of all PLS-DA models was based on the leave-one-out cross validation method via parameters Q2 and R2. The significance of these models was demonstrated by permutation test with 2000 iterations using separation distance and *p*-value < 0.05.

## Results

**FIGURE 1:**
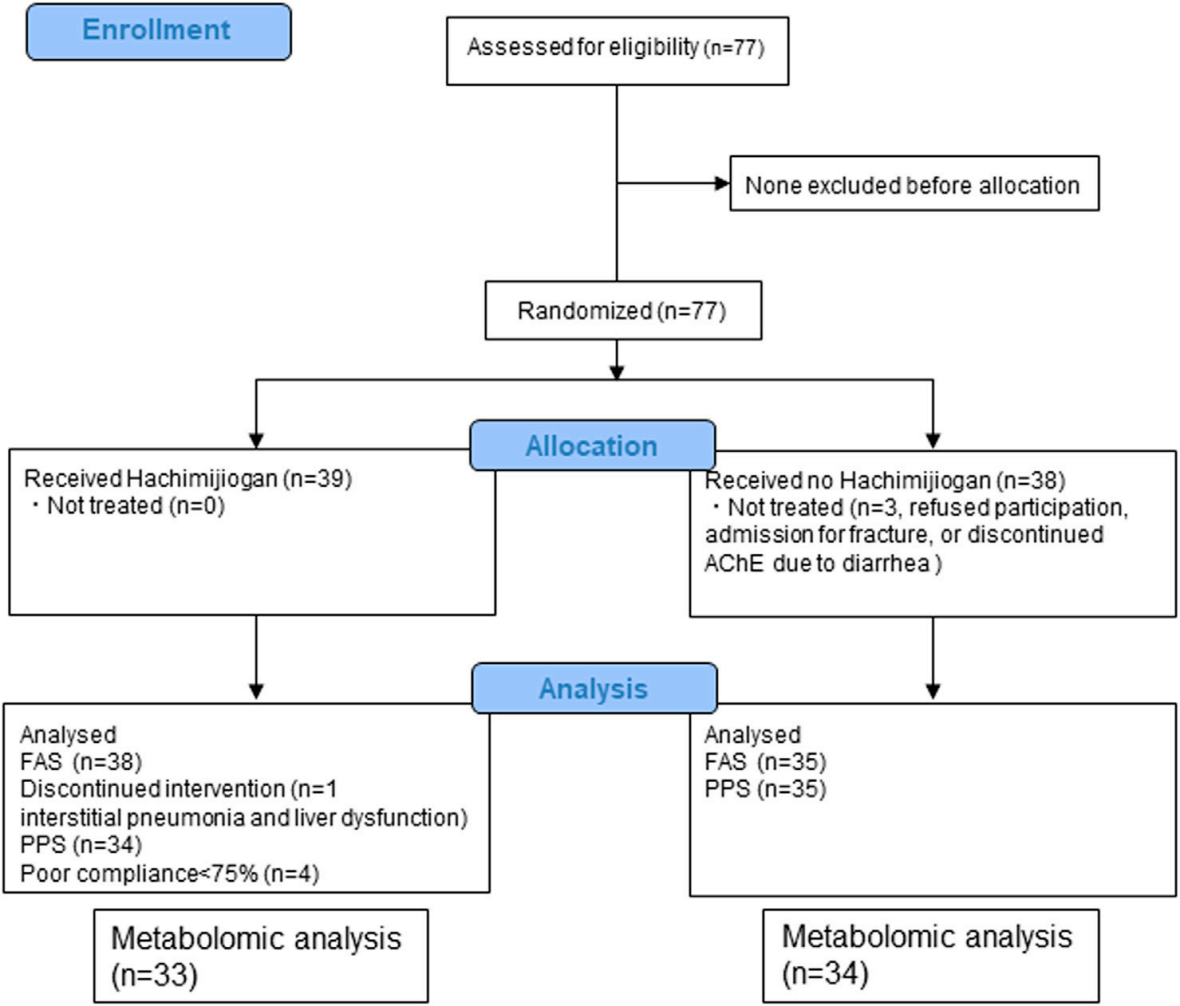
Enrollment, Randomization, and Trial completion.

From among the 77 enrollees, the data of 67 (33 HJG and 34 control) were available for analysis: 2 of the 69 patients analyzed for the primary endpoint were not available for analysis due to hemolysis (Figure 1). No difference was found in the background factors between the HJG and control groups (Table 1).

**TABLE 1:** Baseline characteristics.

	HJG group (*n* = 33)	Control group (*n* = 34)
Sex (male/female)	12/21	14/20
Age, years	75 ± 7.1	76 ± 7.3
Height (cm)	157 ± 9.4	157 ± 7.9
Body weight (kg)	55 ± 11	53 ± 10
Body mass index (Kg/m^2^)	22 ± 3.7	21 ± 3.2
BUN (mg/dL)	17 ± 4.0	17 ± 5.3
Creatinine (mg/dL)	0.83 ± 0.20	0.79 ± 0.26
eGFR (mL/min/1.73 mm^2^)	60 ± 14	67 ± 19
Albumin (g/dL)	4.1 ± 0.3	4.1 ± 0.3
HbA1c (%)	5.9 ± 0.6	6.1 ± 1.0
Systolic Hypertension (mmHg)	131 ± 17	133 ± 16
Diastolic Hypertension (mmHg)	76 ± 13	79 ± 11
Disease duration, years	3.7 ± 2.9	2.8 ± 2.2
Education (university/non university)	10/23	6/28
ADS-Jcog	14 ± 5.4	14 ± 5.7
IADL	5.3 ± 2.5	4.9 ± 2.2
NPI-Q score	4.9 ± 7.5	4.4 ± 8.5
Apathy scale	14 ± 7.4	14 ± 6.5
Hypertension, n (%)	14 (42%)	17 (50%)
Dyslipidemia, n (%)	11 (33%)	13 (38%)
Diabetes mellitus, n (%)	6 (18%)	6 (18%)
Old brain infarction, n (%)	1 (3%)	0 (0%)
AChE inhibitor		
Donepezil	17 (52%)	23 (68%)
Galantamine	10 (30%)	6 (18%)
Rivastigmine	6 (18%)	4 (12%)

### Differential metabolite analysis

We conducted a comprehensive analysis of plasma metabolites to search for HJG biomarkers. The results for the plasma metabolites of male participants at 3 months in the HJG group showed significant change in hippuric acid, DHA, EPA, and phosphatidylcholine (PC) (36_1) and PC (36_2). At 6 months, hippuric acid, phenylacetic acid, suberic acid, tartaric acid, 2-Aminooctanoic acid, and 3-phosphoglyceric acid also showed more significant change in the HJG group than in the control group.

For the female participants of the HJG group at 3 months, suberic acid, tartaric acid and 1,5-anhydro-glucitol showed significant change. They also had significantly greater change of aspartic acid, 1-hexadecanol, 2-hydroxyisovaleric acid, 3-hydroxybutyric acid, and 3-hydroxyisobutyric acid at 6 months.

**FIGURE 2:**
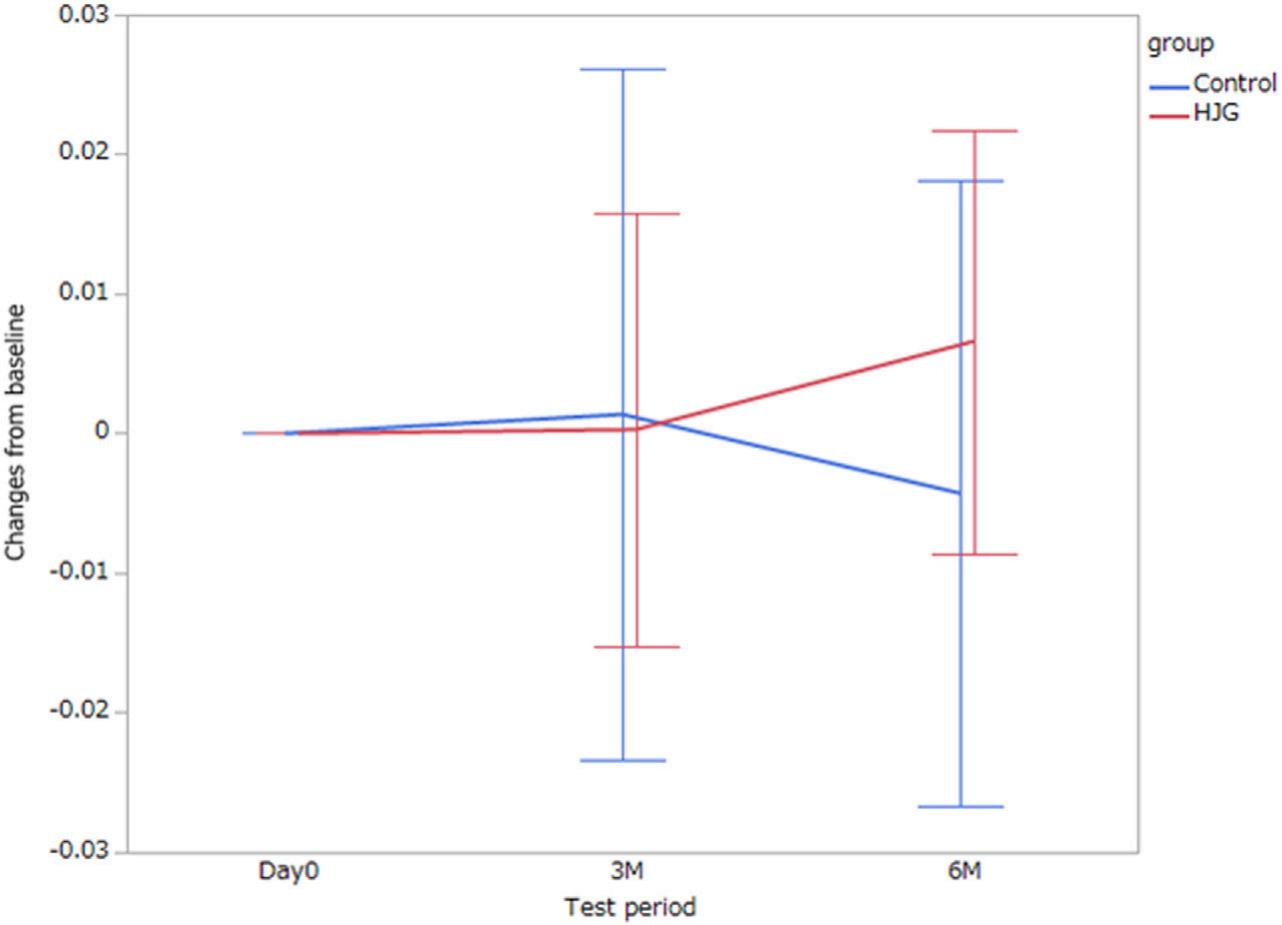
Difference between the HJG and control group female participants in the change of aspartic acid from baseline to 3 and 6 months.

In the comparison of the metabolomics data for which more significant change from baseline was seen in the HJG group than in the control group at each testing point (day0, 3 M, and 6 M), the 3-phosphoglyceric acid of male participants at 6 months was significantly higher in the HJG group (Table 2). For the female participants, aspartic acid was significantly higher in the HJG group than in the control group (Table 3). In the analysis of aspartic acid, the difference in the change from baseline to 6 months of the HJG and control groups was 0.0099 (95% CI, 0.0015 to 0.0184 *p* = 0.0223) (Figure 2).

**TABLE 2:** Comparison of metabolites that had a significant difference in change from baseline (Male).

	Control			HJG		
	Day0	3M	6M	Day0	3M	6M
2-Aminooctanoic acid	0.003693 ± 0.001384	0.003932 ± 0.001711	0.003640 ± 0.001457	0.003912 ± 0.001962	0.004848 ± 0.002956	0.005183 ± 0.003472
3-Phosphoglyceric acid	0.000644 ± 0.000649	0.000552 ± 0.000312	0.000469 ± 0.001940	0.000870 ± 0.000529	0.000745 ± 0.000367	0.000815 ± 0.000418*
DHA	23.0969 ± 14.7251	16.6165 ± 10.3592	19.7715 ± 8.12547	23.0022 ± 14.5089	2 8.3068 ± 16.0475*	23.8425 ± 14.9368
EPA	3.87960 ± 2.96857	2.77862 ± 2.22006	3.14813 ± 1.49982	4.73024 ± 3.63317	6.02772 ± 3.99360*	5.36306 ± 3.849780
Hippuric acid	0.008413 ± 0.004493	0.006756 ± 0.005127	0.006799 ± 0.00464	0.008294 ± 0.009689	0.014966 ± 0.012421*	0.010992 ± 0.009915
PC (36_1)	29525625 ± 7838875.2	29992108 ± 10080484	29016862 ± 70877071.6	26260834 ± 11234604	32087620 ± 18430237	26650192 ± 8319154.6
PC (36_2)	183062364 ± 34245385	179692379 ± 38287164	169556757 ± 33394758	156467692 ± 45534033	183376700 ± 5684525	168844522 ± 50887932
Phenylacetic acid	0.001992 ± 0.001410	0.001965 ± 0.001427	0.001614 ± 0.001306	0.002155 ± 0.001514	0.002357 ± 0.001448	0.002563 ± 0.001682
Suberic acid	0.000955 ± 0.000267	0.000950 ± 0.000235	0.000872 ± 0.000190	0.000953 ± 0.000266	0.000964 ± 0.000187	0.000994 ± 0.000140
Tartaric acid	0.003790 ± 0.005934	0.002819 ± 0.002584	0.003532 ± 0.005427	0.002014 ± 0.000288	0.002772 ± 0.001098	0.002598 ± 0.000595

Mean ± SD. *p < 0.05.

**TABLE 3:** Comparison of metabolites that had a significant difference in change from baseline (Female).

	Control			HJG		
	Day0	3M	6M	Day0	3M	6M
Hexadecanol	0.003556 ± 0.001064	0.003724 ± 0.001236	0.003915 ± 0.001074*	0.003719 ± 0.000785	0.003770 ± 0.001004	0.003184 ± 0.000672
1,5-Anhydro-glucitol	0.002644 ± 0.001193	0.002899 ± 0.001379	0.002799 ± 0.001222	0.002580 ± 0.000993	0.002469 ± 0.000914	0.002679 ± 0.001137
2-Hydroxyisovaleric acid	0.023122 ± 0.008545*	0.022005 ± 0.007776	0.020061 ± 0.006961	0.017151 ± 0.005780	0.017675 ± 0.007805	0.019717 ± 0.008704
3-Hydroxybutyric acid	0.721135 ± 0.569033*	0.544997 ± 0.429438	0.501202 ± 0.359687″	0.326616 ± 0.341714	0.498769 ± 0.817871	0.191881 ± 0.156353
3-Hydroxyisobutyric acid	0.139914 ± 0.08404″	0.112181 ± 0.063638	0.105465 ± 0.056151″	0.079633 ± 0.053163	0.105273 ± 0.119125	0.061700 ± 0.028088
Aspartic acid	0.039730 ± 0.020842	0.041106 ± 0.016687	0.035451 ± 0.010251	0.038523 ± 0.014260	0.038809 ± 0.012114	0.045122 ± 0.016428*
Suberic acid	0.000945 ± 0.000313	0.000916 ± 0.000200	0.000981 ± 0.000207	0.000936 ± 0.000233	0.001029 ± 0.000188	0.000929 ± 0.000237
Tartaric acid	0.002395 ± 0.001089	0.001995 ± 0.000225	0.002935 ± 0.003280	0.002494 ± 0.001064	0.002797 ± 0.001203″	0.004170 ± 0.003245

Mean ± SD *p < 0.05, ″*p* < 0.01.

### Visualization of metabolites

**FIGURE 3:**
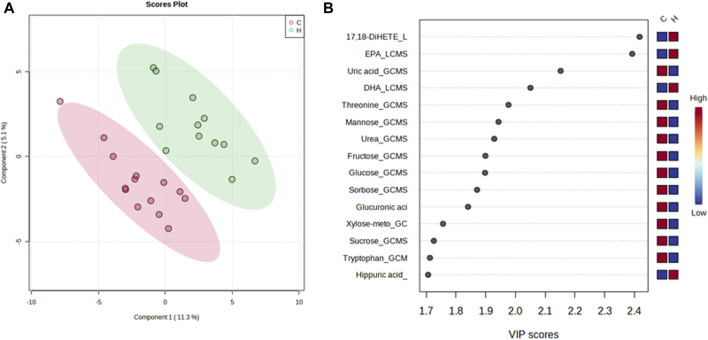
Partial least squares-discriminate analysis (PLS-DA) plot of the differential metabolites of the HJG and control group male participants and their importance in the projection (VIP) values at 3 months. **(A)** Score plot for PLS-DA of the HJG (H, green dots) (*n* = 12) and control groups (C, red dots) (*n* = 14). **(B)** The highest VIP values for the 1st component of PLS-DA. The blue to red color shift denotes low to high plasma metabolite concentrations.

**FIGURE 4:**
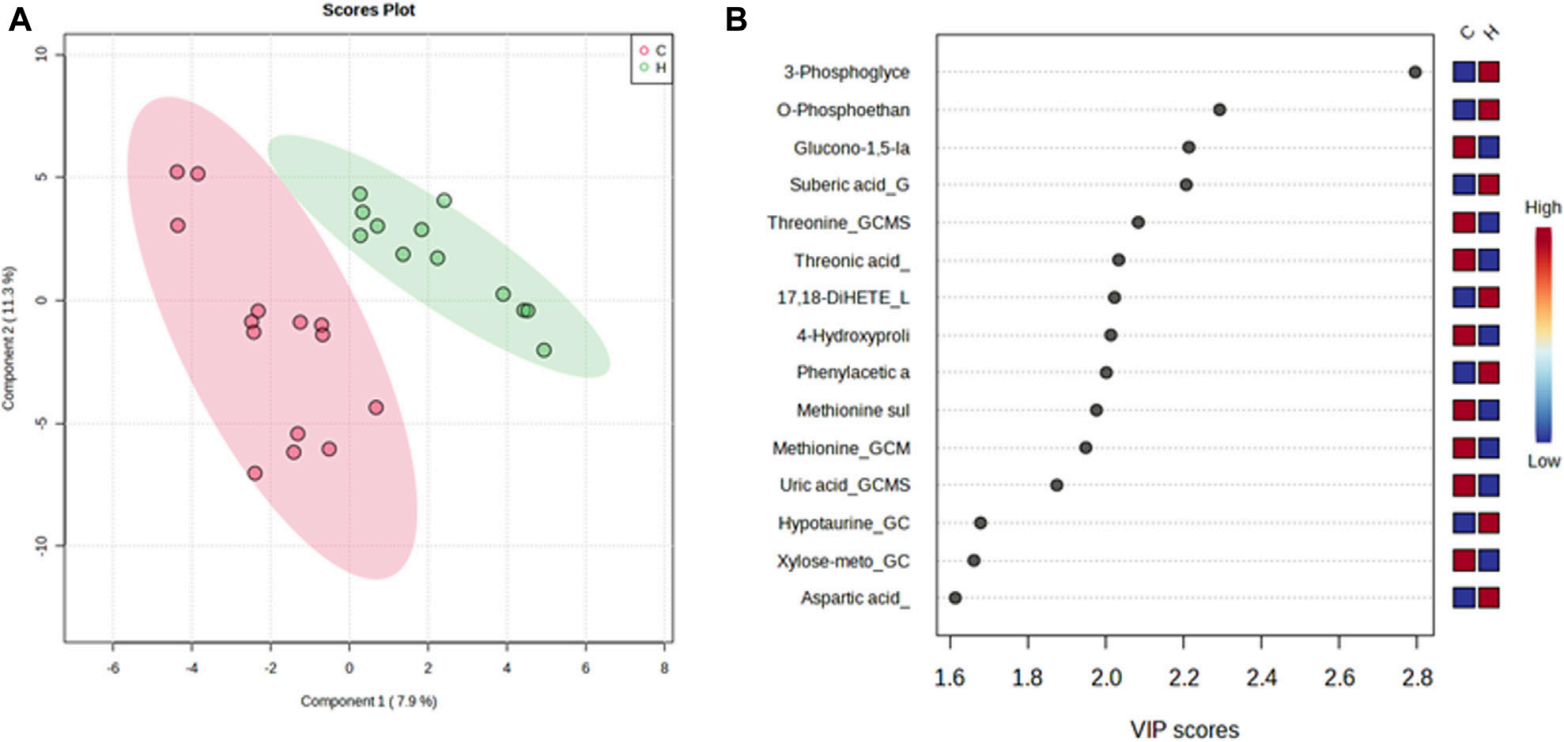
PLS-DA plot of differential metabolites and their corresponding VIP values for male participants at 6 months. **(A)** Score plot of the PLS-DA of the HJG (H, green dots) (*n* = 12) and control groups (C, red dots) (*n* = 14). **(B)** The highest VIP values for the 1st component of PLS-DA. The blue to red color shift denotes low to high plasma metabolite concentrations.

A PLS-DA model with unit variance scaling was used to visualize the differences in the metabolites of the HJG and Control groups and to determine which metabolites can be used to distinguish between the two groups, As shown in Figures 3, 4, plots of the PLS-DA score values of components 1 and 2 demonstrated visible clustering and clear separation between the HJG and control groups. Moreover, the VIP (Variable Importance in Projection) score of the PLS-DA analysis of male participants revealed significantly greater increases in 17,18-DHETE, EPA, DHA, and hippuric acid levels after HJG administration for 3 months than were seen in the control group. 3-phosphoglyce, O-phosphoethan, suberic acid, 17,18-DHETE, phenylacetic acid, hypotaurine, and aspartic acid had a significantly greater increase at 6 months.

**FIGURE 5:**
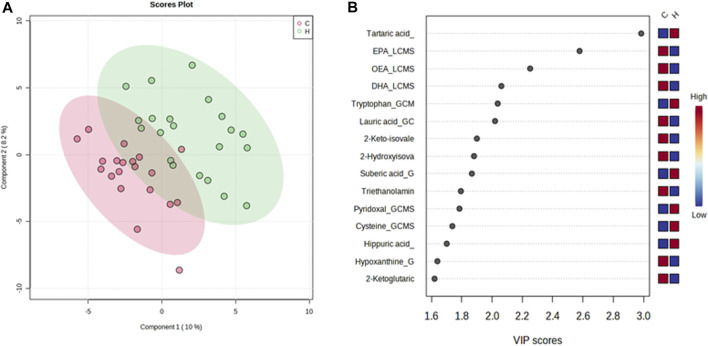
PLS-DA plot of differential metabolites and their corresponding VIP values for the female participants at 3 months. **(A)** Score plot of the PLS-DA of the HJG (H, green dots) (*n* = 21) and control groups (C, red dots) (*n* = 20). **(B)** The highest VIP values for the 1st component of PLS-DA. The blue to red color shift denotes low to high plasma metabolite concentrations.

**FIGURE 6:**
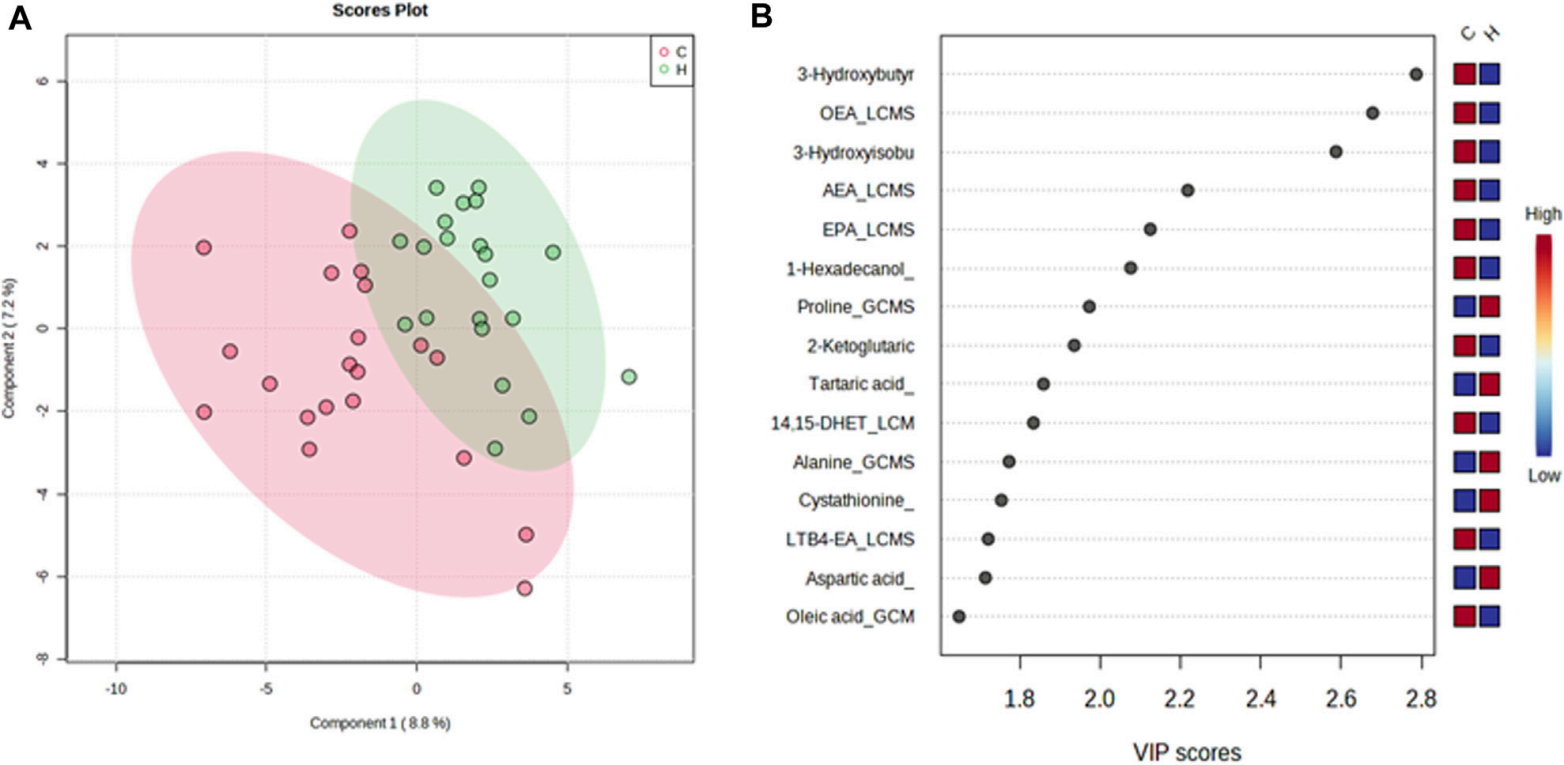
PLS-DA plot of differential metabolites and their corresponding VIP values for the female participants at 6 months. **(A)** Score plot of the PLS-DA of the HJG (H, green dots) (*n* = 21) and control groups (C, red dots) (*n* = 20). **(B)** The highest VIP values for the 1st component of PLS-DA. The blue to red color shift denotes low to high plasma metabolite concentrations.

In contrast, the VIP score of the PLS-DA analysis of female participants revealed a significantly greater increase in tartaric acid, tryptophan, suberic acid, pyridoxal, cysteine, and hippuric acid after HJG administration for 3 months, compared with the control group, and that proline, tartaric acid, alanine, cystathionine, and aspartic acid had a significantly greater increase at 6 months (Figures 5, 6).

## Discussion

This is the first study to use metabolomic analysis to investigate the mechanism(s) of the action of HJG in a clinical study of dementia. The results of univariate and metabolomic analysis showed a significantly greater elevation of aspartic acid after 6 months of HJG in female subjects when compared to controls. These results indicate that an increase in aspartic acid is an important mechanism in the action of HJG on cognitive function. Because this part of the analysis used the same female patients as were used for the primary endpoint, the significantly greater improvement in cognitive function at 6 months of these female patients treated with HJG (the difference in change from baseline at 6 months for female participants was 2.90 (90% CI, 0.09 to 5.71, *p* = 0.090) was shown not to be due to a synergistic effect with AchEI: it was the effect of HJG alone (Kainuma et al., 2022).

Aspartic acid is involved in the development of nerves and their activity in the central nervous system. It has been reported that the concentration of aspartic acid in blood and cerebrospinal fluid was decreased in an AD group compared to a normal group (D'Aniello et al., 2005; Olazarán et al., 2015). In their AD group, aspartic acid was also decreased in the brain, and a decrease in aspartic acid was reported to be correlated with the severity of AD (Kwo-On-Yuen et al., 1994). Of the many reports that use metabolome analysis of early stage AD (Chouraki et al., 2017; Lin et al., 2019a; Orešič, et al., 2011; Shao et al., 2020; Ozaki et al., 2022; Peña-Bautista et al., 2019), some have concluded that aspartic acid is useful for diagnosis (Huo et al., 2020; Olazarán et al., 2015; Trushina and Mielke, 2014; Wilkins and Trushina, 2018). These findings indicate that aspartic acid plays an important role in the maintenance of cognitive function. The results of our study indicate that HJG causes an increase of aspartic acid in the blood and a subsequent increase in aspartic acid in the brain, thereby suppressing the progress of cognitive function.

We previously reported in an *in vitro* study that HJG improves cognitive function through the phosphorylation of CREB (Kubota et al., 2017). Aspartic acid acts as a neurotransmitter and increases cAMP as a second messenger after stimulating receptors (D'Aniello et al., 2011.). Because cAMP is upstream of CREB phosphorylation, an aspartic acid-induced cAMP increase may induce CREB phosphorylation. The results of our study indicate that the increase in aspartic acid by HJG is involved in the phosphorylation of CREB. On the other hand, because aspartic acid in PC12 cells has been reported to increase during neuronal differentiation of PC12 cells (Sano et al., 2018), the increase in aspartic acid may be a product of HJG-induced neurite outgrowth.

Metabolomic analysis of donepezil has been done to examine lipid metabolism, but no significant findings were obtained, and there are no reports of amino acid changes (Wan et al., 2020). There are also no reports on the use of metabolomics for responder analysis of dementia drugs other than donepezil. In this respect, responder analysis of HJG based on metabolome analysis, as used in this study is important, thus our results indicate that aspartic acid would be useful as a therapeutic drug for dementia.

The drugs used in Kampo medicine are combinations of two or more herbs, thus it is difficult to identify the causative agent in the same way as can be done for Western medicines. Although not detected in the univariate analysis in the present study, metabolomic analysis has shown that, in addition to aspartic acid, proline, tartaric acid, alanine, and cystathionine had significantly greater elevation after 6 months than was seen in our control group. Further, proline, alanine, and cystathionine has been correlated with cognitive decline associated with dementia (Xie et al., 2021; Lin et al., 2019b; Zhang et al., 2022). Therefore, it is possible that these substances may interact with each other to improve cognitive function.

The univariate metabolome analysis of the data of male participants showed significantly higher 3-phosphoglyceric acid in the HJG group. There are no reports of correlation between a change of 3-phosphoglyceric acid and cognitive impairment, but our data indicate that this may be related to the effect of HJG. On the other hand, although AD is more common in female patients and gender differences have been reported in the development of AD (Alzheimer’s Association, 2023; Altmann et al., 2014; Tao et al., 2018), in the metabolome analysis of this study a significantly greater increase in aspartic acid was found in our male participants than in the controls after 6 months of HJG. This indicates that the results for men might be similar to those of women if we had had more male patients. Our experience has shown that patients who have signs of “Kidney^[TM]^ deficiency” other than cognitive impairment who take HJG long term have less AD than patients who take it for shorter periods of time. Further studies are needed to confirm this observation.

There are several limitations to the present study. The sample size available for analysis was small, especially that of men. Also, the peripheral blood-brain relation of amino acid remains unknown. Finally, only Japanese populations were recruited for this study.

## Conclusion

Aspartic acid was the main contributor to the difference between female patients treated with HJG in addition to AChEI and a control group treated with only AChEI. A combination of several metabolites was shown to be related to the mechanism of the effectiveness of HJG for mild AD.

## Data Availability

The raw data supporting the conclusion of this article will be made available by the authors, without undue reservation.

## Abbreviations

AChEI; acetylcholinesterase inhibitor, AD; Alzheimer’s disease, ADAS-Jcog; Alzheimer’s Disease Assessment Scale-cognitive component-Japanese version, CREB; cAMP response element binding prote, HJG; Hachimijiogan, RED Cap; Research electronical data capture.
